# Pleistocene South American native ungulates (Notoungulata and Litopterna) of the historical Roth collections in Switzerland, from the Pampean Region of Argentina

**DOI:** 10.1186/s13358-023-00291-5

**Published:** 2023-10-06

**Authors:** Juan D. Carrillo, Hans P. Püschel

**Affiliations:** 1 Department of Biology, University of Fribourg, and Swiss Institute of Bioinformatics, Chemin du Musée 10, Fribourg, Switzerland; 2 Red Paleontológica U-Chile, Departamento de Biología, Facultad de Ciencias, Universidad de Chile, Santiago, Chile

**Keywords:** Pleistocene, Argentina, Santiago Roth, Palaeontological collections, Switzerland, Notoungulata, Litopterna, *Mesotherium*, *Toxodon*, *Macrauchenia*, Pleistoceno, Argentina, Santiago Roth, Colecciones paleontológicas, Suiza, Notoungulata, Litopterna, *Mesotherium*, *Toxodon*, *Macrauchenia*

## Abstract

The fossil collections made by early explorers in South America have been fundamental to reveal the past diversity of extinct mammals and unravel their evolutionary history. One important early explorer in South America was the Swiss-Argentine palaeontologist Kaspar Jacob Roth, known as Santiago Roth (1850, Herisau, Switzerland-1924, Buenos Aires, Argentina), who made significant collections of fossil mammals that are housed in museums in Europe and Argentina. The important collections of Roth in Switzerland include iconic Pleistocene megafauna from the Pampean Region (Argentina). The palaeontological significance of the Pampean Region relies on its abundant record of fossil vertebrates that documents diversity dynamics and paleoenvironmental change in southern South America, serving as the basis for the South American biostratigraphical scale of the late Neogene and Quaternary. The South American native ungulates (SANUs) were hoofed placental mammals that radiated in South America. The clades Notoungulata and Litopterna include, among others, the last representatives of SANUs megafauna in the continent. We revise and describe for the first time the SANUs specimens from the Pampean Region of the Roth collections in Switzerland. The collections include two species of notoungulates (*Toxodon* cf. *T. platensis* and *Mesotherium cristatum*) and one litoptern species (*Macrauchenia patachonica*). The occurrences are restricted to the early and middle Pleistocene (pre-Lujanian Stages/Ages). Although the SANUs diversity in the Roth collections is low in comparison with other groups (e.g., xenarthrans), some of the specimens are very complete, including skulls and postcranial remains. The completeness of the *Ma. patachonica* material allows an update and reinterpretation of some of the details of the dentition and the postcranial skeleton of this iconic species. In addition to its historical importance, the SANU specimens from the Roth collections provide important information to study the paleobiology and evolution of South American megafauna and evaluate hypotheses about their extinction in the continent.

## Resumen

Las colecciones de fósiles realizadas por los primeros exploradores en América del Sur han sido fundamentales para revelar la diversidad pasada de los mamíferos sudamericanos y desentrañar su historia evolutiva. Un importante explorador en América del Sur fue el paleontólogo suizo-argentino Kaspar Jacob Roth, conocido como Santiago Roth (1850, Herisau, Suiza-1924, Buenos Aires, Argentina), quien realizó importantes colecciones de mamíferos fósiles que se encuentran en museos de Europa y Argentina. Las colecciones de Roth en Suiza incluyen la icónica megafauna del Pleistoceno de la Región Pampeana (Argentina). La importancia paleontológica de la Región Pampeana se basa en su abundante registro de vertebrados fósiles que documenta la diversidad y el cambio paleoambiental en el sur de Sudamérica, sirviendo como base para la escala bioestratigráfica sudamericana del Neógeno tardío y Cuaternario. Los ungulados nativos sudamericanos (SANUs, por sus siglas en inglés) eran mamíferos placentarios con pezuñas que radiaron en Sudamérica. Los clados Notoungulata y Litopterna incluyen, entre otros, los últimos representantes de la megafauna de SANUs en el continente. En este trabajo, revisamos y describimos por primera vez los SANUs de la Región Pampeana de las colecciónes de Roth en Suiza. Las colecciónes incluyen dos especies de notungulados (*Toxodon* cf. *T. platensis* y *Mesotherium cristatum*) y una especie de litopterna (*Macrauchenia patachonica*). Las ocurrencias están restringidas al Pleistoceno temprano y medio (Pisos/Edades anteriores al Lujanense). Aunque la diversidad de SANU en las colecciónes Roth es baja en comparación con otros grupos (por ejemplo, xenarthros), algunos de los especímenes son muy completos, e incluyen cráneos y restos poscraneales. Lo completo del material de *Ma. patachonica* permite una actualización y reinterpretación de algunos de los detalles de la dentición y el esqueleto poscraneal de esta icónica especie. Además de su importancia histórica, los SANUs de la colección Roth brindan información importante para estudiar la paleobiología y evolución de la megafauna sudamericana y evaluar hipótesis sobre su extinción en el continente.

## Introduction

The “splendid isolation” and evolutionary history of South American mammals have fascinated palaeomammalogists for more than a century (Croft, 2016; Croft et al., 2020; Patterson & Pascual, 1968; Paula Couto, 1979; Simpson, 1980). The collections of fossil mammals made by early explorers in South America during the 19th and beginning of the 20th centuries have been fundamental to reveal the past diversity of South American mammals and unravel their evolutionary history (Defler, 2019; Fariña et al., 2013; Hatcher, 1985; Vizcaíno et al., 2017). These collections continue to be studied, some even recently highlighted for the first time (e.g., Carrillo-Briceño et al., 2016, 2021; Zurita-Altamirano et al., 2019), and have great historical and scientific value (Vizcaíno et al., 2017). One of the most important early fossil collectors in Argentina was the Swiss-Argentine palaeontologist Kaspar Jacob Roth, known as Santiago Roth (1850–1924), who made significant collections of fossil mammals that are housed in museums in Europe and Argentina (Bond, 1999a; Hansen, 2019; Sánchez-Villagra et al., 2023; Voglino et al., 2023).

Roth conducted numerous surveys in the Pampean Region of Argentina (Sánchez-Villagra et al., 2023; Voglino, 2020; Voglino et al., 2023) and published monographs and catalogues on the mammal fauna, geology and biostratigraphy of the region (e.g., Roth, 1888, 1889, 1904, 1921). Roth sold part of his collection to institutions in Denmark and Switzerland (Hansen, 2019), and today, the specimens are housed in the Zoological Museum of the University of Copenhagen (Hansen, 2019), the Natural History Museum of Geneva, the Cantonal Museum of Geology of the University of Lausanne, and the Palaeontological Institute and Museum of the University of Zurich (Voglino et al., 2023). The important collections of Santiago Roth in Switzerland include iconic Pleistocene megafauna from the Pampean Region, represented by a rich collection of giant glyptodonts and ground sloths, gomphotheres, and native ungulates (Carrillo-Briceño et al., 2023; Christen et al., 2023; Le Verger, 2023; Püschel & Martinelli, 2023; Roth, 1889).

The informal name of South American native ungulates (SANUs) includes several clades of hoofed placental mammals that radiated in South America during the Cenozoic (e.g., Croft et al., 2020 and references cited there). Notoungulata and Litopterna are the two SANU clades with the highest taxonomic diversity and longest temporal range, with a fossil record extending until the Late Pleistocene (Croft et al., 2020). Santiago Roth made a major contribution to the study of SANU evolution by defining the clade Notoungulata based on the anatomy of temporal bones (Roth, 1903).

Although the interrelationships between Notoungulata and Litopterna are not yet fully resolved, there have been important advances in the systematics of these clades (e.g., Billet, 2011; Carrillo & Asher, 2017; Carrillo et al., 2023; Cifelli, 1993; Deraco & García-López, 2016; McGrath et al., 2020b; Püschel et al., 2023; Shockey et al., 2022; Soria, 2001; Vera, 2015; Vera et al., 2019). Notoungulata includes two major sub-clades, Toxodontia (toxodonts and allies) and Typotheria, both of which have representatives that survived until the Late Pleistocene (Billet, 2011; Croft et al., 2020; Deraco & García-López, 2016). The two most diverse subclades within Litopterna are Proterotheriidae and Macraucheniidae, which also survived until the Late Pleistocene (Carrillo et al., 2023; Forasiepi et al., 2016; McGrath et al., 2020a; Püschel et al., 2023; Schmidt, 2015; Villafañe et al., 2006). Moreover, the Late Pleistocene Pampean record of *Toxodon* Owen, 1837 (Notoungulata) and *Macrauchenia* Owen, 1838 (Litopterna) provided key data that helped to clarify the phylogenetic relationships of these two extinct clades within placentals. Molecular data recovered from *Toxodon* and *Macrauchenia* supports a close relationship of notoungulates and litopterns with perissodactyls (Buckley, 2015; Welker et al., 2015; Westbury et al., 2017).

The palaeontological significance of the Pampean Region that attracted the attention of Roth and other explorers, relies on its abundant record of fossil vertebrates that documents diversity dynamics and paleoenvironmental change in southern South America and serves as the basis for the South American biochronological scale of the late Neogene and Quaternary (Cione & Tonni, 1995; Cione et al., 2015; Domingo et al., 2020; Gasparini et al., 2023; Pascual & Jaureguizar, 1990; Prado et al., 2021; Prevosti et al., 2021). The Pleistocene was a period of high mammal diversity in the Pampean Region, in particular megafauna, that was affected by paleoenvironmental changes (Le Verger, 2023; Prado et al., 2001; Prado et al., 2021). The SANUs megafauna survived in the Pampean Region until the Late Pleistocene/Holocene boundary (Prado et al., 2015), and its extinction in the region was likely the result of a synergy of paleoclimatic changes and anthropogenic impacts (Barnosky & Lindsey, 2010; Cione et al., 2003).

Here, we revise for the first time the Pleistocene SANUs specimens from the Pampean Region of the Roth collections in Geneva and Zurich. We provide a list of the referred material with identifications to the species level with anatomical descriptions, comments on the completeness of the specimens, and stratigraphic information when possible.

## Materials and methods

We studied the SANUs specimens from the Roth collection housed in the Natural History Museum of Geneva (MHNG) and the specimens from the 5th catalogue of the fossils collected by Roth that were sold to the canton of Zurich (Roth, 1889) and currently housed at the Palaeontological Institute and Museum of the University of Zurich (PIMUZ). Descriptions were based on direct observations and current images from museum collections of relevant specimens. We revised their taxonomic identification according to the most recent studies for each taxon. We follow the criteria of Püschel et al. (2023) for dealing with the dental formula of litopterns and other SANUs. We follow Vera et al. (2019) on the dental terminology of Typotheria and Madden (1990) on Toxodontia. We follow Nessov et al. (1998) and Lobo et al. (2017) for the dental terminology of macraucheniids, although with some modifications and additions explained in the text and figures that update in a few instances the terminology previously used for this group. Measurements were taken in millimetres, either manually with a calliper, or digitally using Fiji (ImageJ v2.1.0) (Schindelin et al., 2012), to the nearest two decimal places. We followed the Nomina Anatomica Veterinaria (International Committee on Veterinary Gross Anatomical Nomenclature, 2017) for the anatomical terminology, and Bengston (1988) on the use of open nomenclature. Three-dimensional surface models of selected specimens described here are available at Sketchfab (https://sketchfab.com/PIMUZ).

**Fig. 1:**
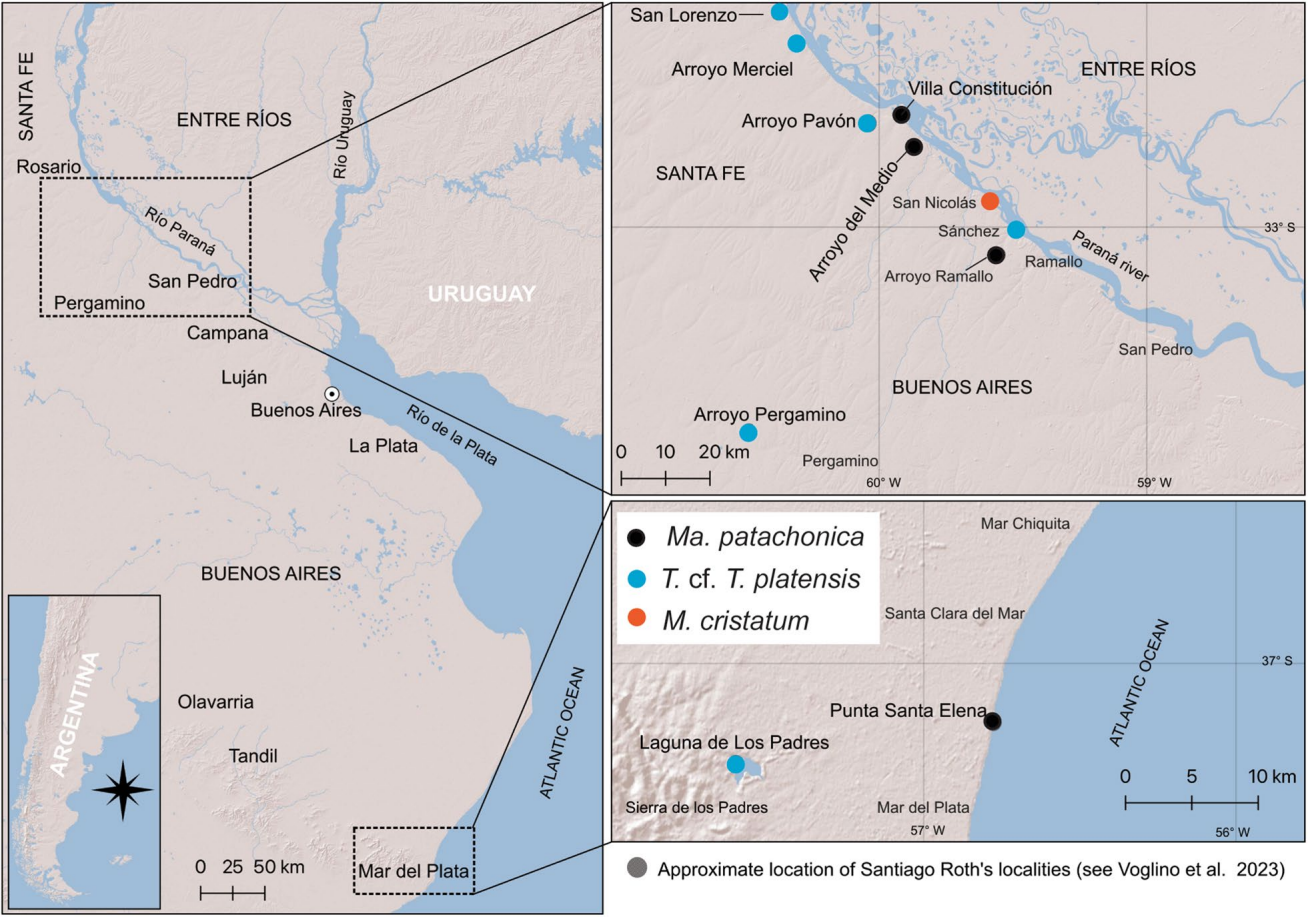
Geographic provenance of the SANUs specimens of the Roth collection from the Pampean Region, Argentina

All the specimens are from the Pampean Region in the Santa Fe and Buenos Aires Provinces, Argentina (Fig. 1). The precise stratigraphic provenance of the specimens is not known. Roth (1889) used the strata “Pampéen inférieur” (Lower Pampean), “Pampéen moyen” (Intermediate Pampean) and “Pampéen supérieur” (Upper Pampean). When possible, we provide the original strata provenance of each specimen as indicated by Roth in Table 1. Recent revisions on the chronostratigraphy and geochronology of the Pampean Region have considered that the age of the sediments, where Roth made his collections roughly correspond to the Ensenadan, Bonaerian, and Lujanian Ages/Stages (Table 1) (see Cione & Tonni, 1995; Cione et al., 2015; Voglino, 2020; Voglino et al., 2023; Le Verger, 2023; Fernández-Monescillo et al., 2023d and references therein).

**Table 1:** Provenance and age of the SANU specimens of the Roth collection from the Pampean Region, Argentina

Species	Collection number	Roth Catalog	Material	Locality	Strata (Roth, 1889)	Estimated Age/Stage (Voglino et al., 2023)
*Mesotherium cristatum*	PIMUZ A/V 467	131	Partial cranium and mandible	San Nicolás	Intermediate Pampean	late Ensenadan–Bonaerian
	PIMUZ A/V 4133	40	Partial mandible	San Nicolás	Lower Pampean	Ensenadan
*Toxodon* cf.* T. platensis*	PIMUZ A/V 4163	225	Partial mandible	Sánchez	Lower Pampean	Ensenadan
	PIMUZ A/V 4199	250	Isolated P2 and P3	Arroyo Pavón	Intermediate Pampean	late Ensenadan–Bonaerian
	PIMUZ A/V 4210	36	Partial mandible	Arroyo Pergamino	Intermediate Pampean	late Ensenadan–Bonaerian
	PIMUZ A/V 4216	98	Right femur and partial pelvis	Arroyo Pavón	Intermediate Pampean	late Ensenadan–Bonaerian
	PIMUZ A/V 4233	205	Isolated m2	Arroyo Pergamino	Intermediate Pampean	late Ensenadan–Bonaerian
	PIMUZ A/V 4245	212	Isolated P4	San Lorenzo	Intermediate Pampean	late Ensenadan–Bonaerian
	PIMUZ A/V 4290	62	Left ulna	Arroyo Merciel	Intermediate Pampean	late Ensenadan–Bonaerian
	PIMUZ A/V 5697	33	Partial cranium	Arroyo Pergamino	Intermediate Pampean	late Ensenadan–Bonaerian
	MHNG GEPI V3665	–	Partial skeleton	Unknown locality	Pampean	Unknown
	MHNG GEPI V5791	–	Two dental fragments	Laguna de los Padres	Pampean	Unknown
	MHNG GEPI V5792	–	Right I2	Bahía Blanca	Pampean	Unknown
	MHNG GEPI V5793	–	Fragmentary scapulae and three vertebrae	Unknown	Pampean	Unknown
	MHNG GEPI V5794	–	Left humerus	Unknown	Pampean	Unknown
*Macrauchenia patachonica*	PIMUZ A/V 4118	248	Isolated right M2	Villa Constitución	Intermediate Pampean	late Ensenadan–Bonaerian
	PIMUZ A/V 4119	109	Isolated left M2	Arroyo Ramallo	Intermediate Pampean	late Ensenadan–Bonaerian
	PIMUZ A/V 5700	223	Partial skeleton	Arroyo del Medio	Intermediate Pampean	late Ensenadan–Bonaerian
	MHNG GEPI V3659	–	Partial cranium, mandible and scapula	Unknown	Pampean	Unknown
	MHNG GEPI V3660	–	Partial mandible	Punta Santa Elena	Pampean	Unknown
	MHNG GEPI V3661	–	Partial mandible	Punta Santa Elena	Pampean	Unknown

*Institutional Abbreviations*. **MACN-PV**, Museo Argentino de Ciencias Naturales “Bernardino Rivadavia”, Colección Nacional de Paleontología Vertebrados, Buenos Aires, Argentina; **MLP**, Museo de La Plata, La Plata, Argentina; **MHNG GEPI,** Muséum d’histoire naturelle, Geneva, Switzerland. **MNHN.F**, Muséum national d’Histoire Naturelle, Palaeontological collection, Paris, France; **PIMUZ A/V**, Palaeontological Institute and Museum of the University of Zurich, Zurich, Switzerland; **YPM VPPU**, Yale Peabody Museum, Vertebrate Palaeontology Princeton University collections, New Haven, USA.

*Other abbreviations*. **I/i**, upper and lower incisor; **ka**, thousand years ago; **M/m**, upper and lower molar; **P/p**, upper and lower premolar; **SANUs**, South American native ungulates.

## Systematic palaeontology

Mammalia Linnaeus, 1758

Eutheria Huxley, 1880

Panperissodactyla Welker et al., 2015

Notoungulata Roth, 1903

Typotheria Zittel, 1893

Mesotheriidae Alston, 1876

*Mesotherium* Serres, 1867

*Mesotherium cristatum* Serres, 1867

* Referred material*. PIMUZ A/V 467, partial skull and mandible (Catalogue No. 5, specimen 131; Roth, 1889; Table 1); PIMUZ A/V 4133, partial mandible (Catalogue No. 5, specimen 40; Roth, 1889; Table 1).

* ﻿﻿Provenance*. PIMUZ A/V 467 and PIMUZ A/V 4133 come from San Nicolás, Buenos Aires Province, Argentina. PIMUZ A/V 467 comes from the Intermediate Pampean and PIMUZ A/V 4133 comes from the Lower Pampean (Roth, 1889; Table 1; Fig. 1).

**Fig. 2:**
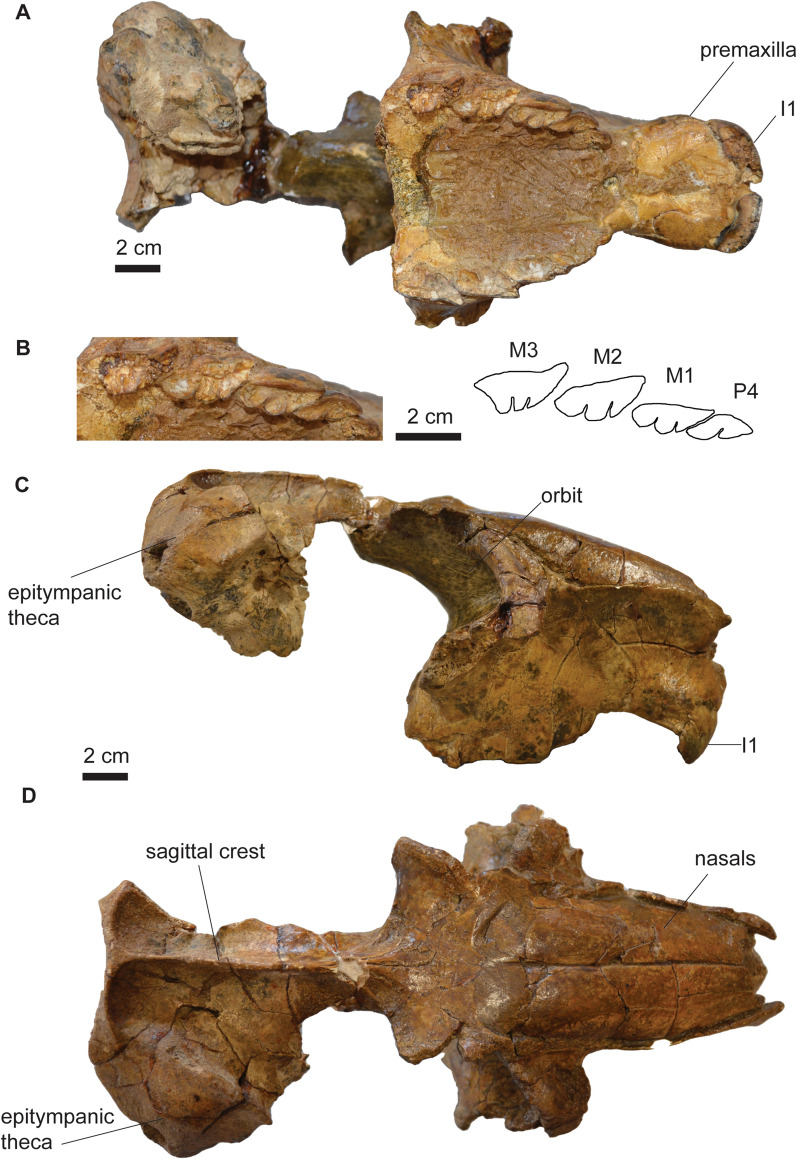
Cranium of *Mesotherium cristatum* (PIMUZ A/V 467). **A** Ventral view. **B** Detail (left) and drawing (right) of the upper right dentition in occlusal view﻿. **C** Lateral view. **D** Dorsal view. Notice the inflated epitympanic theca, the lateral borders of premaxilla diverging rostrally, the mesiodistally elongated I1 and the lateral orientation of the parastyle of M3 (see Fernández-Monescillo et al., 2023b)

**Fig. 3:**
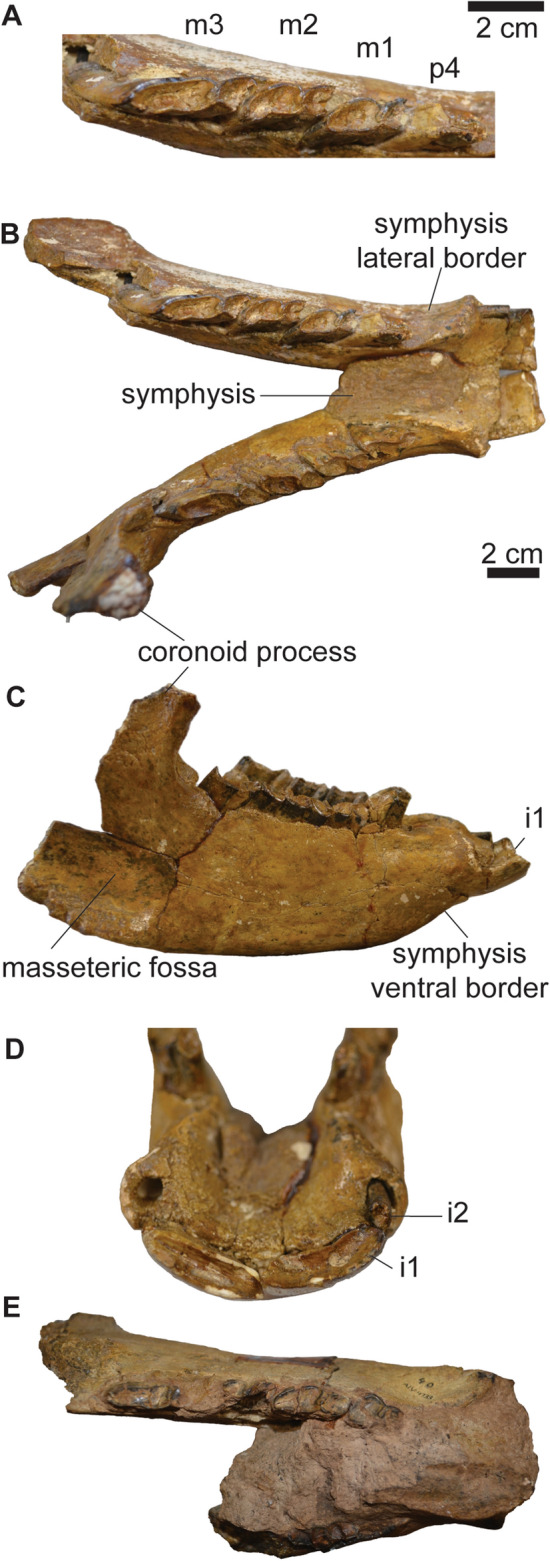
Mandible of *Mesotherium cristatum*. PIMUZ A/V 467 (**A**–**D**). **A** Detail of the left lower dentition in occlusal view. Mandible in **B** dorsal view, **C** lateral, and **D** anterior views. **E** PIMUZ A/V 4133 in dorsal view. Notice the differences in size and occlusal outlines of i1 and i2, the constricted lateral borders and concave ventral border of the symphysis (see Fernández-Monescillo et al., 2023b)

*﻿ ﻿Remarks*. *Mesotherium cristatum* is a common and iconic species in the South American fossil record. In the biostratigraphic scheme of the Pampean Region, it has been traditionally considered as a guide taxon of the Ensenadan Stage/Age (Cione & Tonni, 1995, 1999; Cione et al., 2015; Gasparini et al., 2023). Recently, the distribution of *M. cristatum* has been revised (Bond, 1999b; Fernández-Monescillo et al., 2023d), and the last record of the species has been documented at 220 ± 13 ka, which calls for a revision of the biostratigraphy of the Ensenadan Age (Fernández-Monescillo et al., 2023d). The taxonomic history and revisions of *M. cristatum* are complex due to the lack of recognition of ontogenetic variation (sometimes interpreted as inter-generic or intraspecific variation) and misidentification of specimens and taxa defined for Pliocene strata (Fernández-Monescillo et al., 2023b, 2023c; Simpson, 1940). A recent taxonomic revision concluded that variation among Middle and Early Pleistocene mesotheriinaes from the Pampean Region is consistent with ontogenetic and intraspecific variation of a single species (see Fernández-Monescillo et al., 2023b). We refer PIMUZ A/V 467 to *M. cristatum* based on the I1 mesiodistally elongated with a shallow lingual fold (Fig. 2A), the P4 lingually bilobed (Fig. 2B), the rostrally diverged lateral borders of the premaxilla (Fig. 2A), the inflated epitympanic theca (Fig. 2C), the lateral orientation of the parastyle in M3 (Fig. 2B), the outline of i1 nearly rectangular (Fig. 3D), the i1 having approximately five times the length of i2 (Table 2; Fig. 3D), and the mandibular symphysis with constrained lateral borders and concave ventral border (Fernández-Monescillo et al., 2023b). We refer PIMUZ A/V 4133 to *M. cristatum* based on the dental morphology, the constrained lateral border and straight ventral border of the mandibular symphysis (Fernández-Monescillo et al., 2023b). The dental and craniomandibular dimensions of PIMUZ A/V 467 are given in Tables 2 and 3.

**Table 2:** Dental dimensions (in mm) of *Mesotherium cristatum* (PIMUZ A/V 467) of the Roth collection in Zurich

	I1	P3	P4	M1	M2	M3
Maximum length	10.6	11.6	19.2	22.9		
Maximum width	24.6	5.9	7.7	10.5		
	i1	i2	p4	m1	m2	m3
Maximum length	22.5	4.2	15.8	18.7	21.3	29.0
Maximum width	6.9	5.2	7.6	8.1	8.0	7.7

**Table 3:** Cranium and mandible dimensions (in mm) of *Mesotherium cristatum* (PIMUZ A/V 467) of the Roth collection in Zurich. Measurements follow Cerdeño et al. (2012). *Estimated

Cranium	
Total length*	270
Nasal length*	120
Anterior nasal width	26.6
Posterior nasal width	52.2
Ventral length of premaxilla	35.8
Ventral width of premaxilla	39.6
Maxilla height at the level of P3	66.5
Length P3–M3	83.6
Mandible	
Maximum length*	180
Height horizontal ramus at p4–m1	46.0
Height horizontal ramus behind m3	62.2
Length p4–m1	75.9

*Description*. The upper dentition of PIMUZ A/V 467 is poorly preserved, but nevertheless some important diagnostic features can be observed (Fig. 2). The I1 is mesiodistally elongated, and it has a broad and shallow lingual fold, which is more clearly visible on the left I1 (Fig. 2A). The P3 is present, but the crown is damaged and details of the crown morphology cannot be seen (Fig. 2A, B). The P4 is lingually bilobed, with a mesiolabially oriented lingual sulcus (Fig. 2B). The ectoloph of the upper molars is oriented labially. In M1–2 the ectoloph is straight, whereas in M3, the ectoloph is concave with the parastyle strongly projected labially (Fig. 2B) (Fernández-Monescillo et al., 2023b; Patterson, 1952). There are two lingual sulci on M1–3.

The i1 has a nearly rectangular outline (similar longitudinal width) and is about five times longer than the i2 Table (Fig. 3; Table 2; see also Fernández-Monescillo et al., 2023a, 2023b). Only the crown of the left i2 is preserved, and it is single rooted and circular in cross section. The crown of the p4 is broken on the talonid, and the trigonid is narrow mesially (Fig. 3A). The m1–2 are similar to each other, with well-defined lingual and labial sulci separating the talonid and the trigonid, and the talonid being mesiodistally longer (Fig. 3A). The m3 is mesiodistally longer than m1–2 (Table 2), with an elongated talonid.

The skull is missing the zygomatic arches, the caudal portion of the palate and most of the basicranium (Fig. 2). The sagittal crest (*crista sagittalis externa*) is well-defined, and the nasals extend caudally until the level of the rostral border of the orbit on dorsal view (Fig. 2D). The skull shows two diagnostic features of *M. cristatum*: (a) the lateral borders of the premaxilla diverging rostrally in ventral view (Fig. 2A; Ameghino, 1918), and (b) the inflated epitympanic theca (Fig. 2C; Fernández-Monescillo, 2023b). The mandible is robust, reaching its maximum height at the level of m3 (Table 3; Fig. 3C). The coronoid process (*processus coronoideus mandibulae*) is high and the masseteric fossa (*fossa masseterica*) is wide. The mandibular symphysis extends caudally until the p4. The symphysis shows diagnostic features of *M. cristatum,* such as the constrained lateral borders, and the straight to concave ventral border (Fernández-Monescillo et al., 2023b).

﻿Toxodontia Owen, 1853

Toxodontidae Gervais, 1847

*Toxodon* Owen, 1837

*Toxodon* cf. *T. platensis* Owen, 1837

*﻿Referred material*. PIMUZ A/V 4163, partial mandible with left m1–3, fragments of left p3–4, alveoli of left p1–2, and right p1–4, and isolated right m1–2 (Catalogue No. 5, specimen 225; Roth, 1889; Table 1); PIMUZ A/V 4199, isolated right P2 and P3 (Catalogue No. 5, specimen 250; Roth, 1889; Table 1); PIMUZ A/V 4210, partial mandible with fragments of right and left m1–3, right p3–4, isolated right p1, fragments of right i1–3, and left i1, i3 (Catalogue No. 5, specimen 36; Roth, 1889; Table 1); PIMUZ A/V 4216 right femur and partial pelvis (Catalogue No 5, specimen 98; Roth, 1889; Table 1); PIMUZ A/V 4233, isolated right m2 (Catalogue No. 5, specimen 205; Roth, 1889; Table 1); PIMUZ A/V 4245, isolated left P4 (Catalogue No. 5, specimen 212; Roth, 1889; Table 1); PIMUZ A/V 4290, left ulna (Catalogue No. 5, specimen 62; Roth, 1889; Table 1); PIMUZ A/V 5697, partial skull (Catalogue No. 5, specimen 33; Roth, 1889; Table 1); MHNG GEPI V3665, partial skeleton, including atlas, axis, thoracic and lumbar vertebrae, ribs, sacrum, scapulae, partial left humerus, partial left ulna, and right femur (Table 1); MHNG GEPI V5791, two dental fragments, one lower premolar, possibly p2, and an upper molar fragment; MHNG GEPI V5792, right I2 (Table 1); MHNG GEPI V5793, fragmentary scapula and three thoracic vertebrae (Table 1), and MHNG GEPI V5794, partial left humerus (Table 1).

*﻿Provenance*: PIMUZ A/V 4163 comes from Sánchez, Buenos Aires Province (Lower Pampean). PIMUZ A/V 4199 and PIMUZ A/V 4216 come from Arroyo Pavón, Santa Fe Province (Intermediate Pampean). PIMUZ A/V 4210, PIMUZ A/V 4233, and PIMUZ A/V 5697 come from Arroyo Pergamino, Buenos Aires Province (Intermediate Pampean). PIMUZ A/V 4245 comes from San Lorenzo, Santa Fe Province (Intermediate Pampean). PIMUZ A/V 4290 comes from Arroyo Merciel (Intermediate Pampean) (Roth, 1889; Table 1; Fig. 1).

*﻿Remarks*. The first documented fossil remains of *Toxodon* were collected by Charles Darwin on the Voyage of the HMS Beagle in the 1830s, and later described by the Richard Owen (Fariña et al., 2013; Fernicola et al., 2009; Owen, 1837, 1838). Previous studies have described the skull and postcrania of *Toxodon*, and several *Toxodon* species have been described for the Pleistocene of the Pampean Region (Ameghino, 1887a; Bond et al., 1995; Liendo Lazarte, 1941; Miño-Boilini et al., 2006; Owen, 1837, 1838; Roth, 1895, 1898). Although there are not recent systematic revisions of the genus *Toxodon*, it has been proposed that only two species are likely valid for the Pleistocene of the Pampean Region, *Toxodon platensis* and the significantly smaller *Toxodon gracilis* Gervais & Ameghino, 1880 (Miño-Boilini et al., 2006)*.* The other species would represent intraspecific and ontogenetic variation of a single species (*T. platensis*) (Miño-Boilini et al., 2006). We refer PIMUZ A/V 4163, 4199, 4233, 4245, 4290, and 5697 to *Toxodon* cf. *T. platensis* based on their size and overall morphology, as some of the described *Toxodon* species have not been formally designated as synonyms of *T. platensis* and following Bengston (1988) on the use of open nomenclature.

**Fig. 4:**
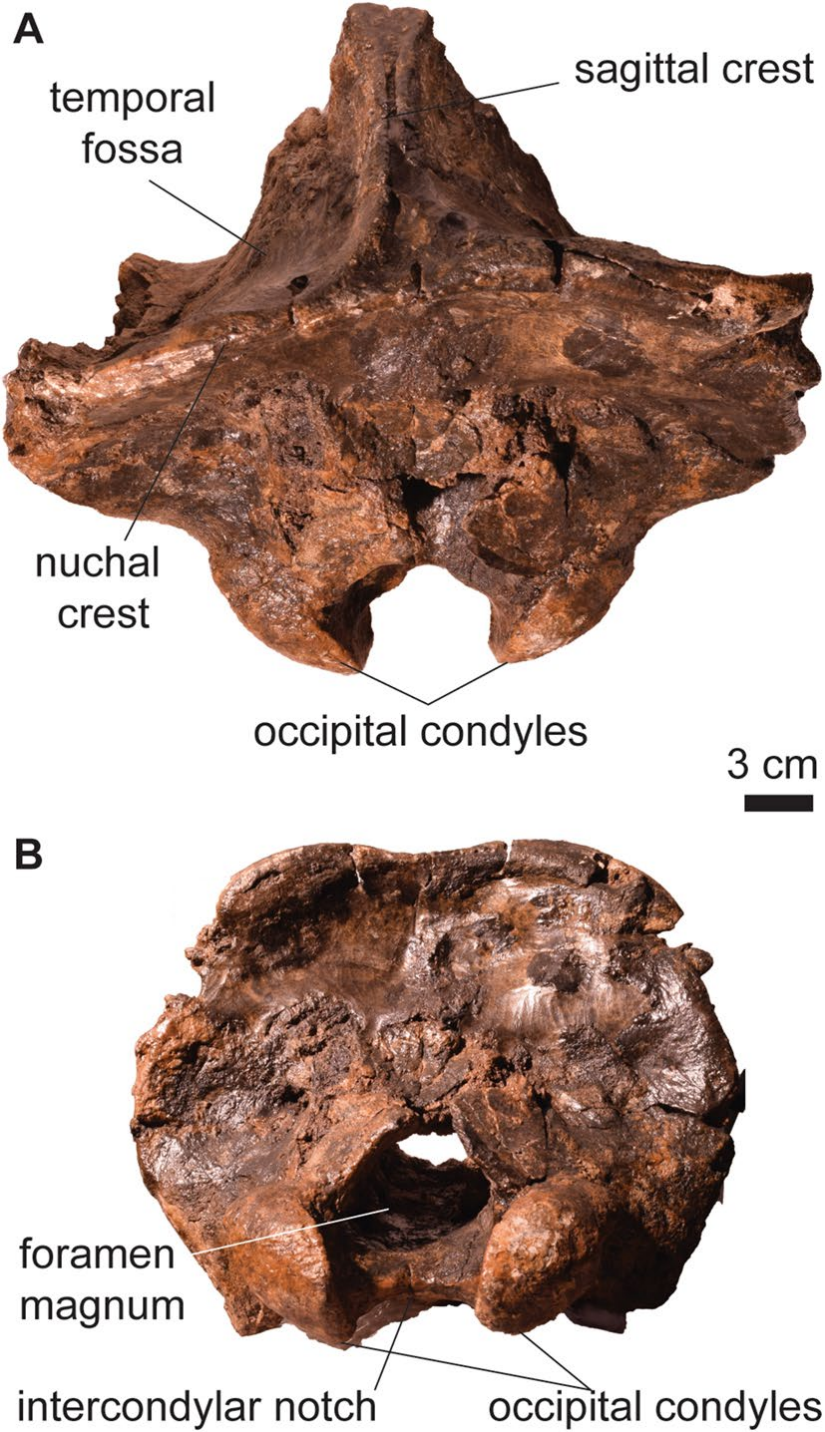
Partial skull of *Toxodon* cf. *T. platensis* (PIMUZ A/V 5697). **A** Caudal portion of the cranium in dorsal view. **B** Occiput in caudal view

*﻿Description*. PIMUZ A/V 5697 preserves only part of the cranium, including the occiput, occipitals, and part of the parietals (Fig. 4; Table 4). The sagittal (*crista sagittalis externa*) and the nuchal crest (*crista nuchae*) are well-developed, defining a wide temporal fossa (*fossa temporalis*) (Fig. 4A). The occipital condyles (*condylus occipitalis*) are large and oval, separated by a wide intercondylar notch (Fig. 4B). PIMUZ A/V 5697 shows no differences with the occipital part of the cranium described for other specimens of *Toxodon* (Roth, 1895, 1898).

**Table 4:** Cranium dimensions (in mm) of *Macrauchenia patachonica* and *Toxodon* cf. *T*. *platensis* of the Roth collection in Zurich

	*Ma. patachonica* (PIMUZ A/V 5700)	*T.* cf. *T. platensis* (PIMUZ A/V 5697)
Maximum width between occipital condyles	101.11	167.76
Maximum posterior dorsoventral height	131.91	

**Fig. 5:**
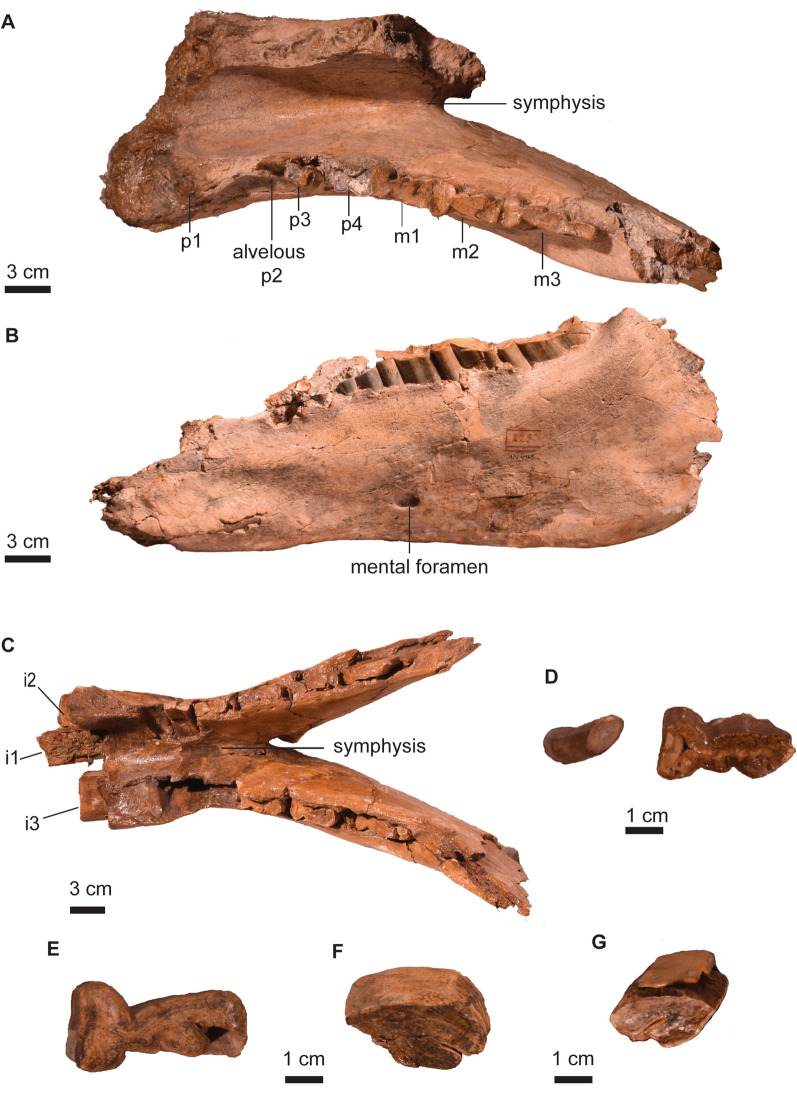
Mandible and dentition of *Toxodon* cf. *T. platensis.* Mandible (PIMUZ A/V 4163) in **A** dorsal and **B** lateral views. **C** Mandible (PIMUZ A/V 4210) in dorsal view. **D** Right p1 (left) and m2 (right) in occlusal view (PIMUZ A/V 4163). **E** Right m2 in occlusal view (PIMUZ A/V 4233). **F** Left P4 in occlusal view (PIMUZ A/V 4245). **G** Right P3 in occlusal view (PIMUZ A/V 4199)

The two partial mandibles (PIMUZ A/V 4163 and 4210) are robust, as in *T. platensis* and unlike in *T. gracilis* (Ameghino, 1887a, 1887b; Miño-Boilini et al., 2006). The mandibular symphysis extends caudally until the level of m1 (Fig. 5A, C). In *Toxodon*, the symphysis extends until the p4 in juveniles and until the m1–2 in adults (Roth, 1898). The mandibular body (*corpus mandibulae*) in PIMUZ A/V 4163 is better preserved than in PIMUZ A/V 4210. The horizontal ramus of the mandible (*ramus mandibulae*) is deep and it has a mental foramen (*formanen mentale*) at the level of m1 (Fig. 5B).

The I2 (MHNG GEPI V5792; Table 5) is developed as a tusk, it is poorly preserved, but two enamel bands are visible, on the mesial, and mesiolingual sides. The P2 (PIMUZ A/V 4199) is rhomboid in occlusal view, with labial and lingual enamel bands (Miño-Boilini et al., 2006). The P3 (PIMUZ A/V 4199) and P4 (PIMUZ A/V 4245) are similar in morphology, but the P4 is larger (Table 5). They have a lingual enamel fold oriented mesiolabially, lingual, and labial enamel bands (Fig. 5F, G). The lower incisors are only partially preserved in PIMUZ A/V 4210 and the base of the labial incisors (i3) are wider than the mesial ones (i1–2) (Fig. 5C), as in *T. platensis* (Ameghino, 1887a; Miño-Boilini et al., 2006; Roth, 1898). Only the crown of the right p1 is preserved, it is simple and oval in occlusal view (Fig. 5D). In the two partial mandibles (PIMUZ A/V 4163 and 4210) only parts of the crowns of p3 and p4 are preserved (Fig. 5A–C). They are elongated mesiodistally in occlusal view. The p4 has a labial groove (Fig. 5A). The lower molars have a buccal enamel fold on the labial side and anterior, meta-entoconid, and ento-hypoconulid folds on the lingual side (Fig. 5A, C–E). The size of the molars increases from m1 to m3 (Table 5). The m3 is elongated distomesially. The dental dimensions of the lower and upper teeth are witrange of known specimens of *T. platensis* (Table 5) (Miño-Boilini et al., 2006).

**Table 5:** Dental dimensions (in mm) of *Toxodon* cf. *T. platensis* and *Macrauchenia patachonica* of the Roth collections in Zurich and Geneva

Specimen	*Toxodon* cf. *T. platensis*						*Ma. patachonica*											
	PIMUZ A/V 4163			MHNG GEPI V5792	PIMUZ A/V 4199	PIMUZ A/V 4245	PIMUZ A/V 4118	PIMUZ A/V 4119	MHNG GEPI V3661		MHNG GEPI V3660		MHNG GEPI V3659					
Tooth	m1	m2	m3	I2	P3	P4	M2	M2	p3	p4	m2	m3	i3	c	P4	M1	M2	dP4 +
Maximum width	17.97	21.46	18	23.5	15.73	20.96	33.31	29.97	14.5	19.43	22.1	24.65	10.65	15.4	27.6	29.8	34.79	29.8
Maximum length	40.95	44.79	61	21.1	26.79	35.83	55.56	50.93	28.1	34.46	46.6	48.74	17.63	22.9	29.4	34	43.66*	45
Crown height							35.08*	38.15*	17.3	16.28	18.9	23.01	12.08*	15.9	25.2	19.8	35.26	35.7

*Estimated. + Molar from a different individual

**Fig. 6:**
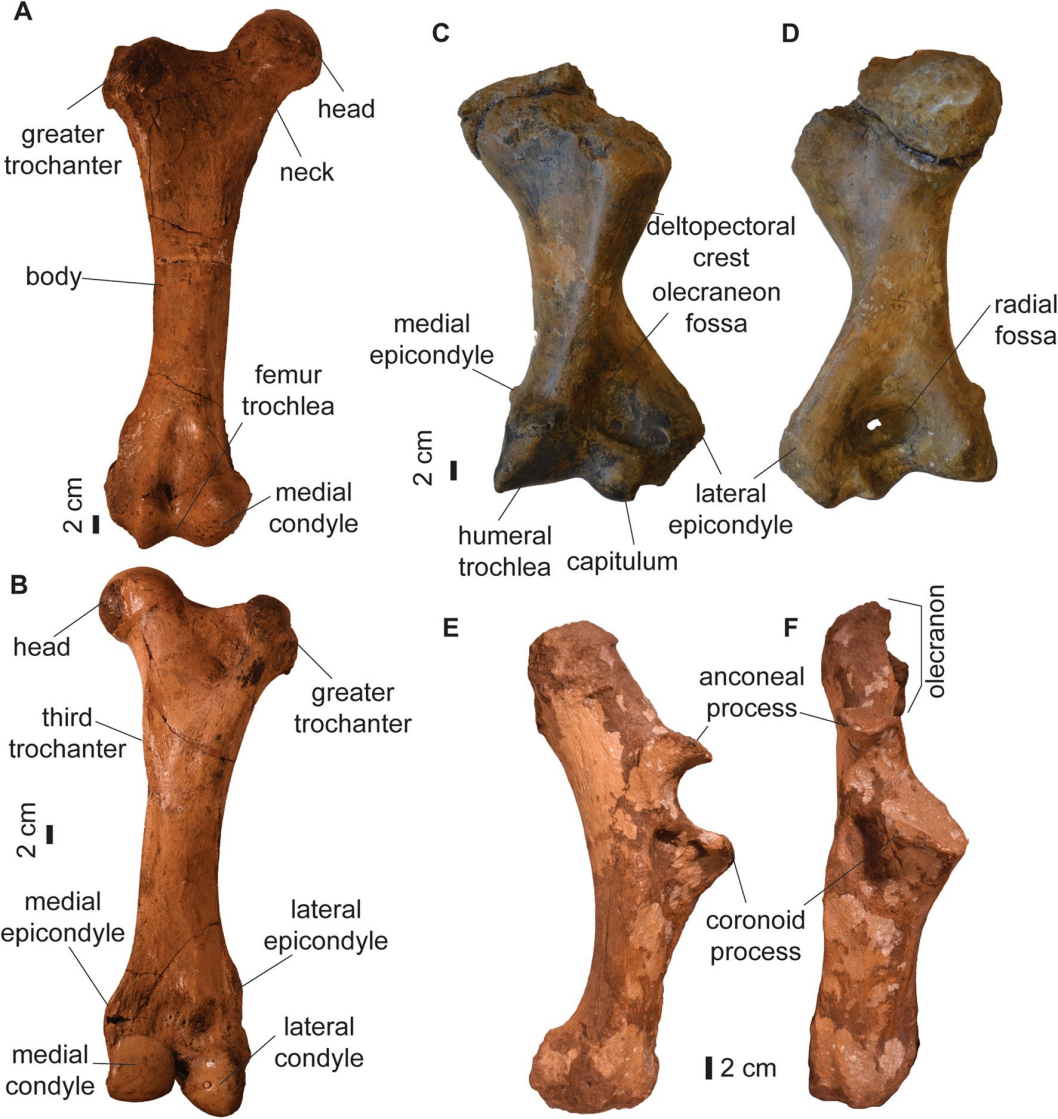
Limb bones of *Toxodon* cf. *T. platensis* from the Roth collections in Zurich and Geneva. Right femur (PIMUZ A/V 4216) in **A** anterior view, and **B** posterior view. Partial left humerus (MHNG GEPI V3665) in **C** anterior view, and **D** posterior view. Left ulna (PIMUZ A/V 4290) in **E** lateral view, and **F** medial view

The limb bones of *Toxodon* have been described in previous works (e.g., Liendo Lazarte, 1941; Miño-Boilini et al., 2006; Roth, 1898), and the specimens of the Roth collections in Zurich and Geneva do not differ on the morphology or dimensions from elements previously described. The two right femora are similar in length (maximum length is 620 mm for MHNG GEPI V3665, and 600 mm for PIMUZ A/V 4216; Fig. 6). The humeri are robust, with a well-developed lateral epicondyle (*epicondylus lateralis*) (Fig. 6). Humeri dimensions are provided in Table 8. The left ulna (PIMUZ A/V 4290) is complete. The total length is 479 mm (Fig. 6), within the range of other *Toxodon* ulnae known (Roth, 1898). The shaft (*corpus ulnae*) is robust and is arched posteriorly (Fig. 6A). The olecranon is large and measures 163 mm. It is straight and longer than the trochlear notch (*incisura trochlearis*), which forms a crescent in lateral view (Fig. 6A). The anconeal process (*processus anconeus*) is large and projects anteriorly. The coronoid process (*processus coronoideus ulnae*) is larger than the anconeal process, and it extends medially (Fig. 6). PIMUZ A/V 4290 shows no differences with other described ulnae of *Toxodon* (Roth, 1898).

Litopterna Ameghino, 1889

Macraucheniidae Gervais, 1855

Macraucheniinae Gervais, 1855

*Macrauchenia* Owen, 1838

*Macrauchenia patachonica* Owen, 1838

*Referred material*. PIMUZ A/V 4118, isolated right M2 with the roots missing (Catalogue No. 5, specimen 248; Roth, 1889; Table 1); PIMUZ A/V 4119, isolated left M2 with the tips of the roots broken (Catalogue No. 5, specimen 109; Roth, 1889; Table 1); PIMUZ A/V 5700, posterior portion of the cranium, complete series of seven cervical vertebrae (atlas, axis, C3–7), five thoracic vertebrae (T1–5), and right scapula and right forelimb, including the humerus, ulna-radius, carpals (scaphoid, lunate, cuneiform, pisiform, trapezium, trapezoid, magnum, and unciform), metacarpals (Mc II, Mc II, and Mc IV), and phalanges (proximal, intermediate, and distal; Catalogue No. 5, specimen 223; Roth, 1889; Table 1); MHNG GEPI V3659, anterior portion of right mandible with extremely worn i2, i3, and c, left fragment of maxilla with P4, M1–2, distal fragment of scapula, and rootless isolated right dP4 from a different individual; MHNG GEPI V3660, partial mandible with left m2–3; MHNG GEPI V3661, right partial mandible with p3–4. In the Zurich Roth collection, there is a right femur and pelvis fragment (PIMUZ A/V 4216) referred by Roth (1889) to *Macrauchenia patachonica* (Catalogue No. 5, specimen 98), which considering its size, proportions and anatomical features does not pertain to this taxon. We refer PIMUZ A/V 4216 to *Toxodon* cf. *T. platensis*.

*Provenance*. PIMUZ A/V 4118 comes from Villa Constitución, Santa Fe Province (Intermediate Pampean), PIMUZ A/V 4119 comes from Arroyo Ramallo, Buenos Aires Province (Intermediate Pampean), and PIMUZ A/V 5700 comes from Arroyo del Medio, Buenos Aires Province (Intermediate Pampean) (Roth, 1889; Table 1; Fig. 1). MHNG GEPI V3659 comes from an unknown locality of the Buenos Aires Province (Pampean), MHNG GEPI V3660 and MHNG GEPI V3661 come from Punta Santa Elena, Buenos Aires Province (Pampean) (Table 1; Fig. 1).

*Remarks*. *Macrauchenia patachonica* was first discovered by Darwin in 1834 in the vicinities of Puerto San Julián, Argentinean Patagonia, and later described by Richard Owen (Fernicola et al., 2009; Owen, 1838). Most of the skeleton of *Ma. patachonica* have been described in previous works (e.g., Burmeister, 1864, 1864–1869; Fernández de Álvarez, 1940; Gervais, 1855; Owen, 1838; Püschel & Martinelli, 2023; Sefve, 1925) or compared with earlier macraucheniids (e.g., Püschel et al., 2023; Scott, 1910), so here we focus only in relevant aspects previously unnoticed of some of the elements, expanding the anatomical knowledge of this iconic species. The craniodental and postcranial dimensions of PIMUZ A/V 5700, PIMUZ A/V 4118, PIMUZ A/V 4119, MHNG GEPI V3659, MHNG GEPI V3660, and MHNG GEPI V3661 are given in Tables 4, 5, 6, 7, 8, 9, and 10.

**Table 6:** Vertebrae dimensions (in mm) of *Macrauchenia patachonica* (PIMUZ A/V 5700) of the Roth collection in Zurich

Vertebrae	C2	C3	C4	C5	C6	C7	T1	T2	T3	T4	T5
Maximum vertebral body length	206.65	217.04	204.55	178.6	110.54	77.28	59.05	67.01	66.08	64.89	61.86
Maximum width at anterior end of vertebral body	**–**	75.85	86.06	78.69	82.7	73.78	123.57	107.12	104.46	109.19*	116.83
Maximum dorsoventral height at anterior end of vertebral body	**–**	47.32	55.91	54.1	57.17	54.49	45.07	45.37	42.37	46.92*	50.75
Maximum width at posterior end of vertebral body	83.32	90.69	88.52	87.84	81.21	112.27	113.89	111.83	109.53	120.2*	105.06
Maximum dorsoventral height at posterior end of vertebral body	51.66	55.07	59.03	54.82	57.04	50.5	50.19	46.28	44.18	29.64*	43.68
Width between posterior transverse processes	103.5	115.4	123.97	125.37*	148.84*	148.72	156.61	141.53	113	120.68*	107.35
Width between postzygapophyses	50.93*	51.5*	50.86	63.28	107*	104.5	86.68	55.82	45.98	33.71	29.08
Width between prezygapophyses	109	–	72.32*	76.37	89.22*	–	103.84*	86.94	51.26	–	36.17*
Width of one anterior articular surface for the atlas	43.51	n/a	n/a	n/a	n/a	n/a	n/a	n/a	n/a	n/a	n/a
Dorsoventral height of one anterior articular surface for the atlas	38.66	n/a	n/a	n/a	n/a	n/a	n/a	n/a	n/a	n/a	n/a
Odontoid process length	27.66	n/a	n/a	n/a	n/a	n/a	n/a	n/a	n/a	n/a	n/a

*Part of the element is missing, so the total dimension is inferred from the complete part (assuming bilateral symmetry). *n/a* not applicable

**Table 7:** Scapula dimensions (in mm) of *Macrauchenia patachonica* of the Roth collections in Zurich and Geneva

Specimen	PIMUZ A/V 5700	MHNG GEPI V3659
Maximum length	400	–
Anteroposterior depth at the scapular notch	103.14	77.14*
Maximum depth at the distal end	127.59	84.69*
Glenoid cavity depth	93.63	70.80*
Glenoid cavity width	66.82	52.77*

*Small portions of the element are missing affecting the measured dimension

**Table 8:** Humerus dimensions (in mm) of *Toxodon* cf. *T. platensis* and *Macrauchenia patachonica* (PIMUZ A/V 5700) of the Roth collections in Zurich and Geneva

Specimen	*Ma. patachonica*	*T.* cf. *T. platensis*	
	PIMUZ A/V 5700	MHNG GEPI V5794	MHNG GEPI V3665
Maximum length	383.35	–	410
Maximum width at proximal end	169.8	–	200
Maximum depth at proximal end	179.84	–	170
Width at the narrowest point (constriction)	74.67	108.5	92.6
Depth at the narrowest point (constriction)	59.29	101.2	79.5
Width at the level of the epicondyles	135.99	220	205
Depth at the level of the epicondyles	83.24	80	72.9
Length of the deltopectoral crest	186.93	–	–

**Table 9:** Metacarpals dimensions (in mm) of *Macrauchenia patachonica* (PIMUZ A/V 5700) of the Roth collection in Zurich

Metacarpals	Mc II	Mc III	Mc IV
Length	208.08	234.89	193.31
Proximal articular depth	53.3	–	43.08
Proximal articular width	37.83	–	–
Distal articular depth	49.94	46.58	52.56
Distal articular width	43.05	52.04	45.8
Diaphysis depth	38.8	–	38.94
Diaphysis width	30.01	35.71	–

**Table 10:** Phalanges dimensions (in mm) of *Macrauchenia patachonica* (PIMUZ A/V 5700) of the Roth collection in Zurich

Phalanges	Phalanx 1, digit II	Phalanx 1, digit III	Phalanx 1, digit IV	Phalanx 2, digit II	Phalanx 2, digit III	Phalanx 2, digit IV	Phalanx 3, digit II	Phalanx 3, digit IV
Length	94.58	77.34	93.13	48.32	53.02	47.13	33.69	
Proximal articular depth	38.49	39.11	39.83	28.20	30.59	29.14	–	–
Proximal articular width	45.7	50.81	43.01	39.30	39.47	39.26	26.39	20.35
Distal articular depth	31.45	32.07	28.83	22.7	20.98	24.54	–	–
Distal articular width	37.41	36.86	36.36	29.33	35.64	29.94	37.17	32.7
Diaphysis depth	27.41	24.39	27.52	–	–	–	n/a	n/a
Diaphysis width	26.89	30.17	27.99	–	–	–	n/a	n/a

*n/a* not applicable

**Fig. 7:**
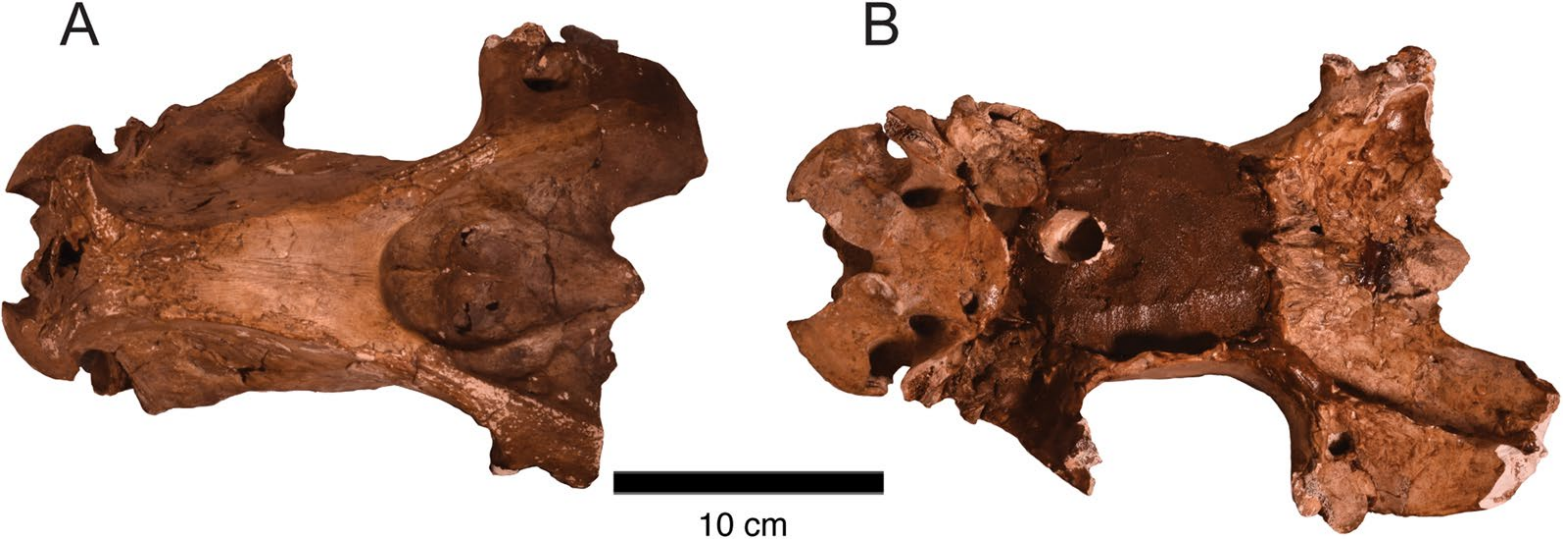
Cranium of *Ma. patachonica* (PIMUZ A/V 5700) in dorsal **A** and ventral **B** views. The cranium (PIMUZ A/V 5700) only preserves its posterior portion and basicranium, and was previously subjected to a poor restoration with plaster which covers some of the cranial structures

*Description*. PIMUZ A/V 5700 includes a fragmentary cranium that preserves the posterior portion, including the basicranium (Fig. 7). Cranial measurements of PIMUZ A/V 5700 are given in Table 4. The skull and dentition of *Ma. patachonica* have been already described in detail by Burmeister (1864, 1864–1869). More recently, some studies focusing on the cranial anatomy, including the petrosal and inner ear anatomy of other litopterns, have used specimens of *Ma. patachonica* in their comparisons revealing previously unknown aspects of macraucheniid’s cranial evolution (Billet et al., 2015; Dozo et al., 2023; Fernández-Monescillo, 2020; Forasiepi et al., 2016).

**Fig. 8:**
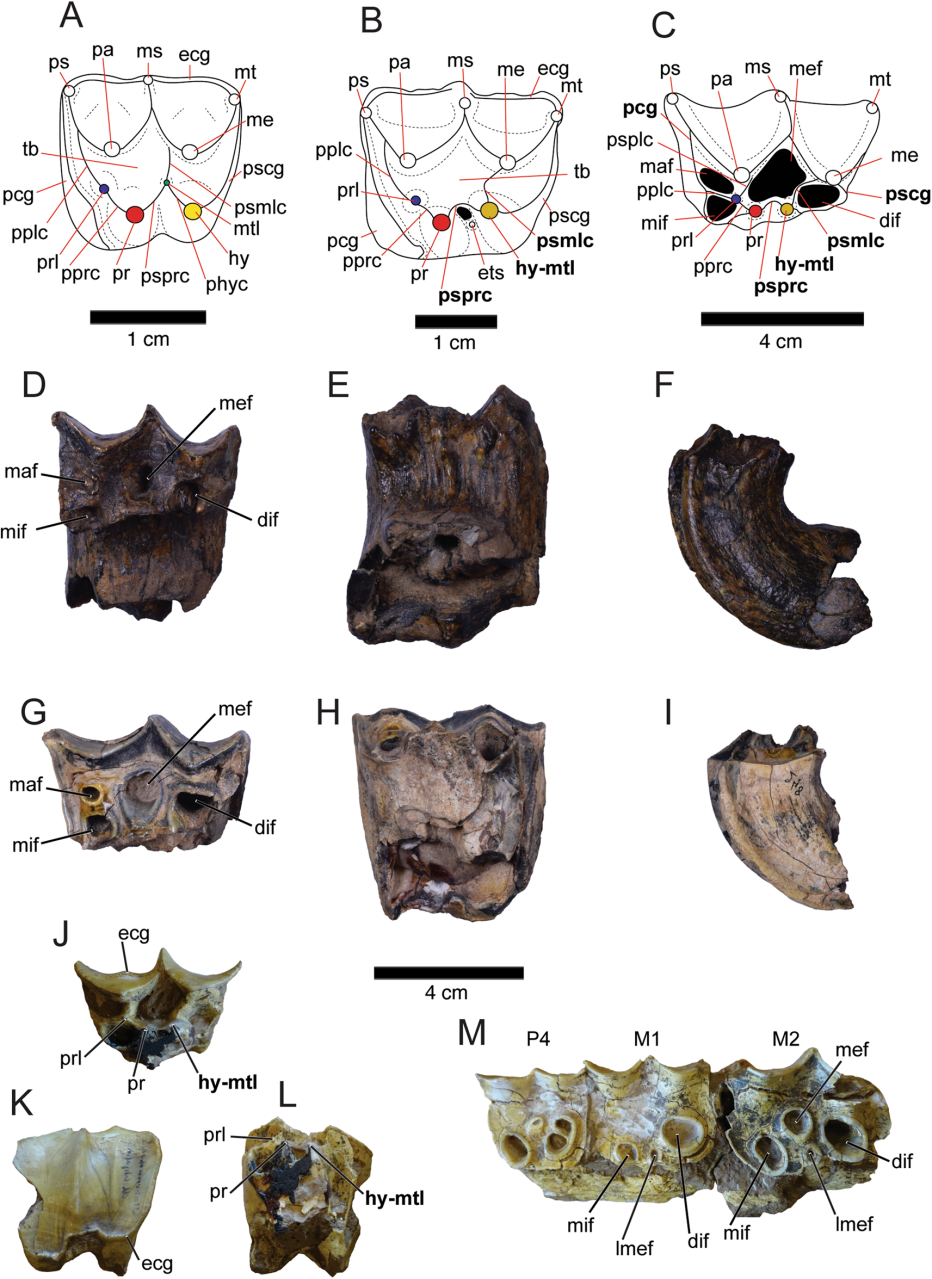
Upper dentition of Macraucheniidae*.* **A** Illustration of a *Cramauchenia normalis* upper molar (M1) indicating the cusps and cristae. **B** Illustration of a *Theosodon lydekkeri* upper molar (M1) indicating the cusps and cristae. **C** Illustration of a *Macrauchenia patachonica* upper molar (M1) indicating the cusps and cristae. **D–F** Relatively unworn left upper molar (M2) of *Ma. patachonica* (PIMUZ A/V 4119**)** in occlusal (**D**), lingual (**E**), and mesial (**F**) views. **G–I.** Worn right upper molar (M2; here mirrored) of *Ma. patachonica* (PIMUZ A/V 4118**)** in occlusal (**G**), lingual (**H**), and mesial (**I**) views. **J–L.** Unworn right deciduous premolar** (**dP4; here mirrored) of *Ma. patachonica* (MHNG GEPI V3659) in occlusal (**J**), buccal (**K**), and lingual (**L**) views. **M** Left maxilla fragment with P4, M1**–**2 of *Ma. patachonica* (MHNG GEPI V3659). The illustration of *C. normalis*’ M1 in **A** of was based on the specimens MLP 83-III-2–1 and MLP 85-V-VII-3-38a. The illustration of *T. lydekkeri*’s M1 in **B** was based on the specimens MACN-A 9269–88 and YPM VPPU 015717. The illustration of *Ma. patachonica*’s M1 in **C** was based on the specimen MACN-PV 11361, among other specimens. In **A–C,** the labels of controversial structures are in bold and relevant cusps are highlighted in different colours: blue for paraconule, red for protocone, green for metaconule, yellow for hypocone, and dark yellow for hypocone–metaconule. Notice that even though the dP4 (**J–L**) and the maxilla fragment with P4, M1**–**2 (**M**) share the same specimen number (MHNG GEPI V3659), these are from different individuals considering their different and incompatible ontogenetic stage (see the extreme wear in the permanent P4 in **M**). *dif* distolingual fossette, *ecg* ectocingulum, *ets* entostyle, *hy* hypocone, *hy-mtl* hypocone–metaconule, *lmef* lingual median fossete, *maf* mesiolabial fossette, *me* metacone, *mef* median fossette, *mif* mesiolingual fossette, *ms* mesostyle, *mt* metastyle, *mtl* metaconule, *pa* paracone, *pcg* precingulum, *pplc* preparaconule crista, *pprc* preprotocrista, *pr* protocone, *prl* paraconule, *ps* parastyle, *pscg* postcingulum, *psmlc* postmetaconule crista, *psplc* postparaconule crista,﻿ *psprc* postprotocrista, *tb* trigon basin

The specimens PIMUZ A/V 4119, PIMUZ A/V 4118, and MHNG GEPI V3659 represent different ontogenetic stages of the upper molars of *Ma. patachonica* (Fig. 8D–M). PIMUZ A/V 4119 and PIMUZ A/V 4118 are M2s with a different wear stage, the former being a relatively unworn left M2 (Fig. 8D–F) and the latter a worn right M2 (Fig. 8G–I). The M2 in MHNG GEPI V3659 (that also preserves the P4–M2 housed in a left maxilla fragment; Fig. 8M), presents higher degree of wear than PIMUZ A/V 4118 and PIMUZ A/V 4119, representing an older individual. However, there is an isolated and unworn deciduous P4 (dP4) stored in MHNG GEPI V collections under the same number (MHNG GEPI V3659; Fig. 8J–L, which it must be from a different and younger individual considering that the left maxilla fragment has a fully erupted P4 with considerable degree of wear (Fig. 8M). The dP4 is almost completely preserved, missing the lingual posteriormost portion of the crown and most of the roots. Overall, the dP4 is similar in morphology to an M1 as is seen in earlier macraucheniids (e.g., *Theosodon lydekkeri* [MACN A 9269-88]) and eutherian mammals in general (Jernvall, 1995). Key differences between the M1 and the dP4 in *Ma. patachonica* are a smaller size, and a considerably lower crown height in the dP4. The dP4 also presents a low ectocingulum or buccal cingulum which is possible to examine at a lower degree of wear (Fig. 8J, K). In contrast, in the permanent dentition the ectocingulum would be only possible to examine in the teeth with the highest degree of wear, considering the crown height and curvature of unworn molars, such as PIMUZ A/V 4119 (Fig. 8D–F). The preservation of PIMUZ A/V 4118 and PIMUZ A/V 4119 does not allow to evaluate the presence of an ectocingulum, as in both specimens the portion of the crown close to the roots and the roots are missing. In *Ma. patachonica*, the M2 is distinguished from M1 by being larger and having a consistently more buccally projected parastyle in comparison with the metastyle, whereas in M1, the parastyle and the metastyle tend to have a subequal buccal projection. The degree of projection of the parastyle in M2 varies with wear and it is easier to evaluate in upper molars in place in the maxilla. Dental measurements of PIMUZ A/V 4119, PIMUZ A/V 4118, MHNG GEPI V3659, and additional specimens that include lower dentition (i.e., MHNG GEPI V3659, MHNG GEPI V3660) are given in Table 5.

The changes of tooth morphology, enamel fossetes, and wear during the ontogeny of *Ma. patachonica* have not been established as it has been in its Pleistocene–Holocene relative *Xenorhinotherium bahiense* Cartelle & Lessa, 1988 (Lobo et al., 2017). However, from observing the upper dentition of several specimens of *Ma. patachonica* in different ontogenetic stages (e.g., MACN-PV 2, MACN-PV 11361, MNHN.F PAM 75, MHNG GEPI V3659), the wear degrees and enamel fossettes patterns of change are overall similar in both taxa. Consequently, in terms of the wear degrees defined by Lobo et al. (2017) for *X. bahiense*, PIMUZ A/V 4119 would be between degrees 1 and 2, and PIMUZ A/V 4118, between degrees 2 and 3, which is also reflected in the crown height of both specimens (Fig. 8D–F, G–I). In *Ma. patachonica*, to these wear degrees, it may be added a degree 6, in which a new lingual median fossette is generated in the position of the protocone (e.g., M2 in MHNG GEPI V3659; Fig. 8M), and also a degree 7, in which the original median fossette disappears (e.g., M1 in MHNG GEPI V3659; Fig. 8M). In addition, it has even been observed in the M1 of a *Ma. patachonica* specimen with extreme wear the disappearance of the lingual median and mesiolingual fossettes (e.g., Sefve, 1925: Fig. 4), which if confirmed in more specimens could be considered a degree 8 of wear.

It is interesting to note that the general morphology and cusps present in unworn M1–2 of *X. bahiense* (Lessa, 1992; Lobo et al., 2017) are extremely similar to those of *Ma. patachonica*. Indeed, Lobo et al. (2017) cusps interpretations for M1–2 of *X. bahiense* in unworn specimens, are mostly similar to our own interpretations of the cusps for M1–2 in *Ma. patachonica* (Fig. 8C). However, the cusp they interpret as a protocone, we interpret it as a paraconule, and in the position of what they described as a protocone–hypocone crest, there is a cusp that we interpret as the actual protocone. The protocone of the M1–2 of *X. bahiense* can be observed more clearly in Lessa (1992: pl 10). The protocone and paraconule are also clearly present in the dP4 of *Ma. patachonica* (MHNG GEPI V3659; Fig. 8J, L). The justification for our identification of the cusps and cristae in the M1–2 of *Ma. patachonica* (Fig. 8C) is given by the M1–2 morphology and cusp configuration of the earlier macraucheniids *Cramauchenia normalis* Ameghino 1902 (Late Oligocene–Early Miocene/Deseadan–Colhuehuapian Ages; Fig. 8A) and *Theosodon lydekkeri* Ameghino, 1887b (Early Miocene/Santacrucian Age; Fig. 8B). *Cramauchenia normalis* has a M1–2 configuration that is relatively easy to interpret as it shares most of the cusps seen in early South American “condylarths” with well-defined conules (Muizon & Cifelli, 2000), presenting, in addition, a well-developed postcingulum-derived “true” hypocone, such as the one present in didolodontid *Didolodus* Ameghino, 1887b (Muizon et al., 2019). It is interesting to note that in *C. normalis* the trigon basin is reduced and shifted mesially, and the metaconule is extremely reduced and very close to the hypocone, being both cusps connected by a crista (prehypocrista; Fig. 8A). In *T. lydekkeri* (and other species of the genus *Theosodon*) the hypocone shifts mesially, occupying the position of the reduced metaconule of *C. normalis*, which could represent a fusion of the metaconule with the hypocone (Soria, 1981; Fig. 8B). Therefore, the prehypocrista is lost in *T. lydekkeri*, and the cristae from the trigon that were connected to the metaconule in *C. normalis* (postprotocrista and postmetaconule crista) are now connected to this hypocone–metaconule cusp. *Macrauchenia patachonica* and also *X. bahiense* keep the same M1–2 cusp configuration displayed by *T. lydekkeri* with an increased hypsodonty, and the presence of enamel fossettes, from which the mesial and distal ones are enclosed by cristae that probably derive from a raised pre and postcingulum (Fig. 8C). Therefore, the lingual cusps that *Ma. patachonica* and *X. bahiense* exhibit in M1–2 (only seen in unworn or slightly worn specimens) going from mesial to distal are: paraconule, protocone, and hypocone–metaconule. The same evolutionary trend occurs in the M3 of macraucheniids.

**Fig. 9:**
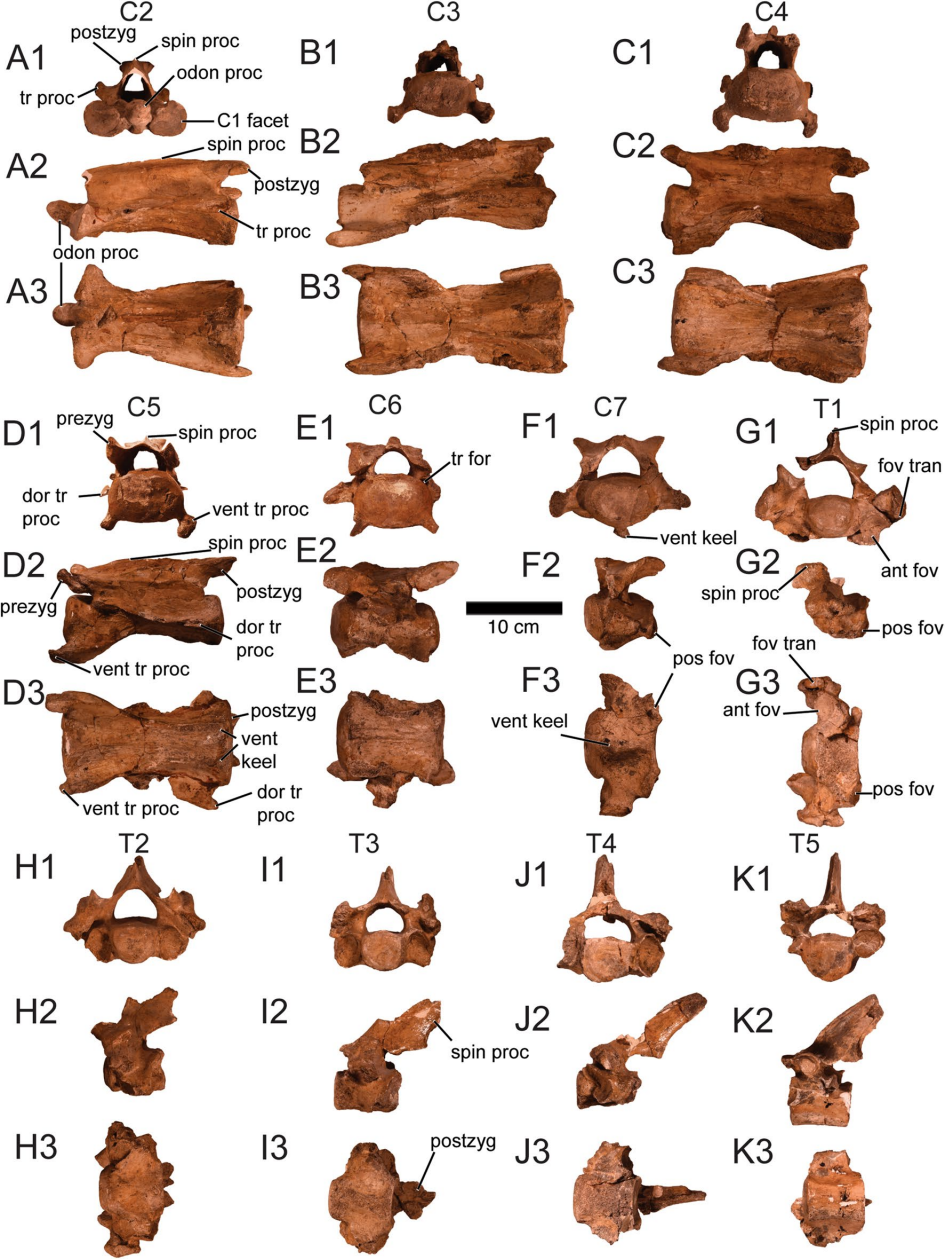
Cervical (C2**–**7) and thoracic (T1**–**5) vertebrae of *Macrauchenia patachonica* (PIMUZ A/V 5700). **A1–3** Second cervical vertebra (C2) in anterior (**A1**), left lateral (**A2**), and ventral (**A3**) views. **B1–3** Third cervical vertebra (C3) in anterior (**B1**), left lateral (**B2**; mirrored), and ventral (**B3**) views. **C1–3** Fourth cervical vertebra (C4) in anterior (**C1**), left lateral (**C2**), and ventral (**C3**) views. **D1–3** Fifth cervical vertebra (C5) in anterior (**D1**), left lateral (**D2**), and ventral (**D3**) views. **E1–3** Sixth cervical vertebra (C6) in anterior (**E1**), left lateral (**E2**; mirrored), and ventral (**E3**) views. **F1–3** Seventh cervical vertebra (C7) in anterior (**F1**), left lateral (**F2**), and ventral (**F3**) views. **G1–3** First thoracic vertebra (T1) in anterior (**G1**), left lateral (**G2**; mirrored), and ventral (**G3**) views. **H1–3** Second thoracic vertebra (T2) in anterior (**H1**), left lateral (**H2**), and ventral (**H3**) views. **I1–3.** Third thoracic vertebra (T3) in anterior (**I1**), left lateral (**I2**), and ventral (**I3**) views. **J1–3.** Fourth thoracic vertebra (T4) in anterior (**J1**), left lateral (**J2**), and ventral (**J3**) views. **K1–3** Fifth thoracic vertebra (T5) in anterior (**K1**), left lateral (**K2**; mirrored), and ventral (**K3**) views. *ant fov* anterior costal fovea, *dor* dorsal, *fov tran* costal fovea of transverse process, *nu can* nutrient canal, *odon proc* odontoid process, *postzyg* postzygapophysis, *pos fov* posterior costal fovea, *prezyg* prezygapophysis,﻿ *spin proc* spinous process, *tr for* transverse foramen, *tr proc* transverse process, *tub* small posterior transverse process tubercle, *vent* ventral

In relation to the vertebrae of *Ma. patachonica*, Burmeister (1864–1869) mostly based on MACN-PV 2, inferred the presence of seven cervical vertebrae, 17 thoracic vertebrae and seven sacral vertebrae. Sánchez-Villagra et al. (2007) mentioned that *Ma. patachonica* has 20–21 thoracolumbar vertebrae based on an unknown MLP specimen, (likely MLP 12-1424, considering that is the most complete *Ma. patachonica* specimen so far described). From examining MLP 12-1424 and PIMUZ A/V 5700 it can be confirmed that *Ma. patachonica* has seven cervical vertebrae. Assuming that the thoracic and lumbar vertebral series is complete in MLP 12-1424, *Ma. patachonica* has 16 thoracic vertebrae and five lumbar vertebrae, making a total of 21 thoracolumbar which is consistent with previous reports (Sánchez‐Villagra et al., 2007). From these, PIMUZ A/V 5700 only preserves the anterior portion (C1–7 and T1–5; Fig. 9). The atlas was previously described wrongly by Burmeister (1864–1869), so it is described in detail and figured correctly for the first time in a different publication of this series which deals specifically with this controversy (Püschel & Martinelli, 2023). Vertebrae measurements of C2–T5 of PIMUZ A/V 5700 are given in Table 6.

The cervical vertebrae are in general elongated (except C6 and C7) with anteroposteriorly long transverse and spinous processes and almost flat faces of their bodies (Fig. 9A1–F3). Previous authors (Burmeister, 1864; Burmeister, 1864–1869; Owen, 1838) described in detail C2–4, and C6. Although the C5 and C7 of *Ma. patachonica* have not been formally described, previous studies have compared their anatomy with those of other macraucheniids, such as *Theosodon* and *Micrauchenia* Püschel et al., 2023 (Püschel et al., 2023; Scott, 1910). The body of C5 is dorsoventrally compressed and ventrally presents two divergent ridges (Fig. 9D1–3). The spinous process of C5 is mediolaterally thin and anteroposteriorly elongated. The transverse process is also anteroposteriorly elongated, but it is divided into two separate sections: a ventral transverse process located in the anterior portion of the vertebra and projects anteriorly from the body, and a dorsal transverse process which extends anteroposteriorly around the dorsoventral midsection of the body. In contrast to C3 and C4, the dorsal transverse process of C5 does not reach the posterior margin of the body, a feature also seen in the C5 of *Theosodon* and *Micrauchenia* (Püschel et al., 2023). As in C2–4 and C6, the transverse foramen (*foramen transversarium*) is on the medial side of the wall of the neural arch. Dorsally the prezygapophyses (*processus articularis cranialis*) and postzygapophyses (*processus articularis caudalis*) form a U-shape and V-shape, respectively. In terms of size, C5 is shorter than C2–4, but longer than C6–7 (Table 6).

In C6 is interesting to note that in PIMUZ A/V 5700, the transverse foramen is located in the midpoint of the wall of the neural arch and the transverse process (Fig. 9E1), in contrast to the specimen illustrated by Burmeister (1864, 1864–1869; MACN-PV 2) in which it is located on the medial side of the wall of the neural arch as in C2–5. We interpret these differences as intraspecific variation. This is interesting, because in *Theosodon,* the transverse foramen perforates the transverse process (Scott, 1910), so PIMUZ A/V 5700 presents an intermediate condition between *Theosodon* and MACN-PV 2. C7 is an anteroposteriorly short cervical vertebra (Fig. 9F1–3). The main differences between C7 with other cervical vertebrae are (a) the absence of a transverse foramen, (b) a continuous and undivided transverse process located at the dorsoventral midpoint of the vertebral body, (c) the presence of a marked ventral keel in the vertebral body located at the mediolateral midpoint, and (d) the presence of posterior costal fovea. In terms of size, C7 is by far the shortest cervical vertebra (Table 6). Detailed comparisons of C7 across different macraucheniids including *Ma. patachonica* (and PIMUZ A/V 5700) have been previously conducted (Püschel et al., 2023).

The thoracic vertebrae of *Ma. patachonica* have a body that in general is notably shorter than the average cervical vertebrae and a more robust spinous process (Fig. 9G1–K3). Burmeister (1864, 1864–1869) described three thoracic vertebrae of *Ma. patachonica* from the Bravard’s collection that he considered to be T6–8, and also T13 and T16 from the MACN collection (later included under the number  MACN-PV 2 ), so the rest of the thoracic vertebrae of this taxon remain undescribed. However, Püschel et al. (2023) gave a detailed description of T2 in *Micrauchenia* that includes comparisons with other macraucheniids, including *Macrauchenia* (PIMUZ A/V 5700). PIMUZ A/V 5700 preserves T1–5 with a variable degree of completeness. From T1 to T5 is possible to see a gradual change in morphology from a more cervical-like thoracic vertebra (T1) to a more typical thoracic vertebra (T5). T1 is extremely similar to C7 in general shape and proportions (Fig. 9G1–3; Table 6), the main differences being (a) the absence of a prominent ventral keel, (b) the presence of anterior costal fovea, (c) a more prominent spinous process and (d) the presence of costal fovea of the transverse process. T2–5 have a more prominent spinous process than T1 and the transverse process is less laterally projected.

As Burmeister (1864–1869) previously observed, there are five gradual morphological changes from the first to the last thoracic vertebrae in many mammals, which apply to *Ma. patachonica*: (a) the anterior vertebrae tend to be larger than the posterior, (b) in the anterior vertebrae, the anterior and posterior costal foveae tend to more ventrally located and larger, but as the vertebrae series progress towards the last thoracic vertebrae, the costal foveae tend to be more dorsally located and smaller, (c) in the anterior vertebrae the transverse process tends to have a more ventral position in relation to the vertebral arch, but as the vertebrae series progress towards the last thoracic vertebrae, the transverse process tends to be more dorsally located, (d) the spinous process tends to change from a more posteriorly bent position in the anterior vertebrae to a more perpendicular position in the posterior vertebrae, and (e) the prezygapophyses and postzygapophyses tend to be overall flat or horizontal in the anterior vertebrae, but as the vertebrae series progress towards the last thoracic vertebrae, the prezygapophyses and postzygapophyses tend to have an angle, being the prezygapophysis somewhat concave and the postzygapophyses somewhat convex.

Comparing the thoracic vertebrae that Burmeister (1864–1869) tentatively referred to T6–8 with the T1–5 in PIMUZ A/V 5700 and the probably complete vertebral series of MLP 12–1424, Burmeister (1864–1869) vertebrae correspond to T4–6 instead of T6–8 considering the length and direction of their spinous processes and the position and size of their anterior and posterior costal foveae.

**Fig. 10:**
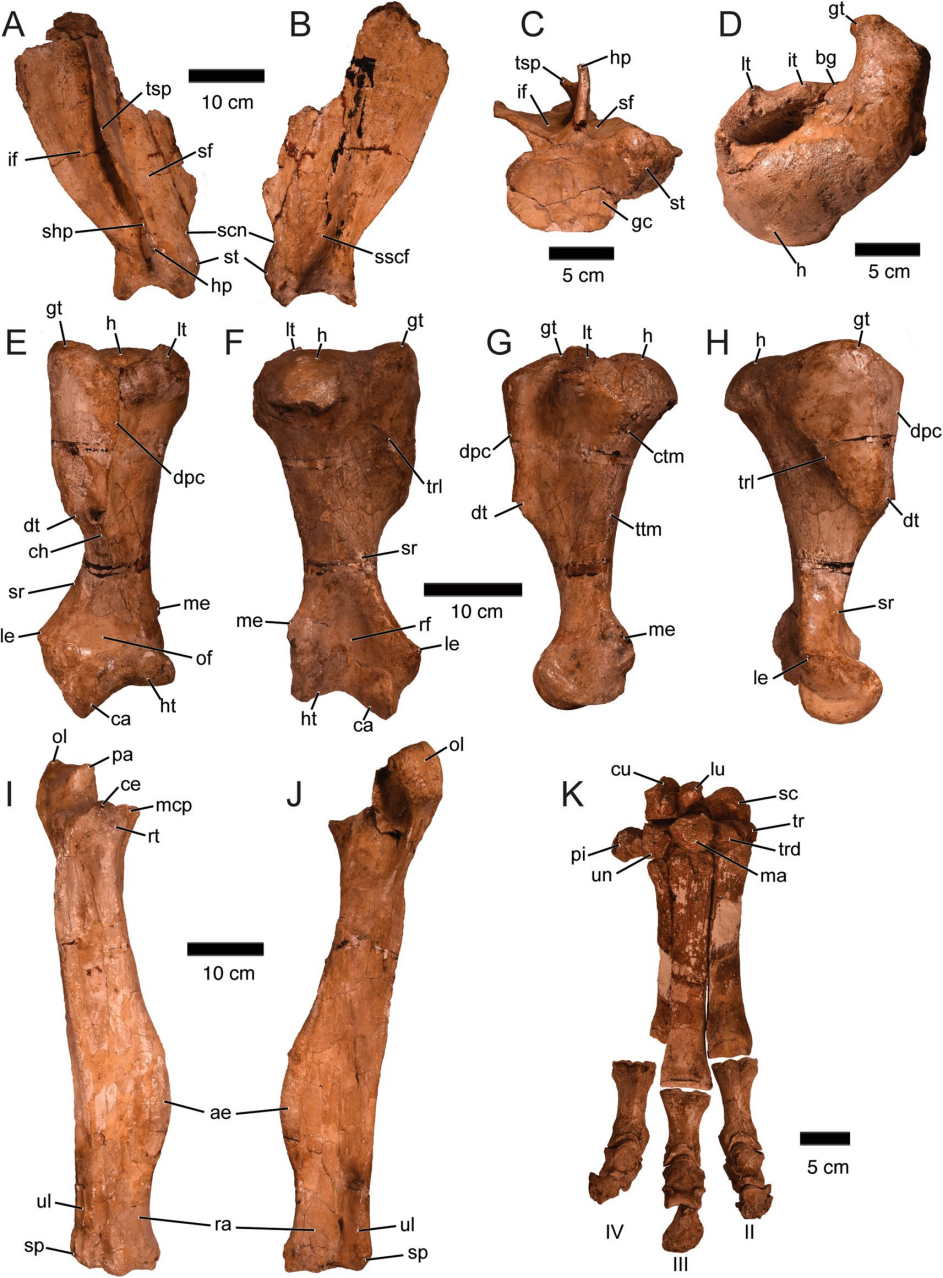
Right forelimb of *Macrauchenia patachonica* (PIMUZ A/V 5700). **A–C** Scapula in lateral (**A**), medial (**B**), and distal (**C**) views. **D–H** Humerus in proximal (**D**), anterior (**E**), posterior (**F**), medial (**G**), and lateral (**H**) views. **I–J** Ulna-radius in anterior (**I**) and posterior (**J**) views. **K** Manus (carpals, metacarpals and phalanges) in anterior view. In** E** the olecranon fossa was restored, so it in PIMUZ is not as deep as it should be. In **K** the carpals and metacarpals were glued together with the different elements in the right position, but in many cases with a wrong orientation (e.g., pisiform and Mc IV). In addition, the ungual phalanx of the third digit is fragmentary, preserving only its distomedial portion. *ae* aliform expansion of the radius, *bg* bicipital groove; ca, capitulum, *ce* capitular eminence, *ch* crest of the humerus, *ctm* crista tuberculi minoris, *cu* cuneiform, *dpc* deltopectoral crest, *dt* deltoid tuberosity,﻿ *gl* glenoid cavity; gt, greater tubercle, *h* head of the humerus, *hp* hamatus process, *ht* humeral trochlea, *if* fossa infraspinata, *it* intermediate tubercle, *le* lateral epicondyle, *lt* lesser tubercle; *lu* lunate, *ma* magnum, *mcp* medial coronoid process (*processus coronoideus medialis*), *me* medial epicondyle, *of* olecranon fossa, *ol* olecranon (*tuber olecrani*), *pa* anconeal process (*processus anconeus*), *pi* pisiform, *ra* radius, *rf* radial fossa; rt, radial or bicipital tuberosity (*tuberositas radii*), *sc* scaphoid, *scn* scapular notch, *sf* fossa supraspinata, *shp* suprahamatus process, *sp* styloid process (*processus styloideus*), *sr* supracondylar ridge, *st* supraglenoid tubercle, *tr* trapezium, *trd* trapezoid,﻿ *trl* tricipital line, *tsp* tuber of the scapular spine,﻿ *ttm* tuberosity of the teres major, *ul* ulna, *un* unciform

The scapula of *Ma. patachonica* has been previously described, particularly its distal portion (Owen, 1838), so what follows is an update of this description considering that the PIMUZ A/V 5700 has a more complete scapula (only missing part of the anterodorsal portion and parts of the scapular spine [*spina scapulae*]; Fig. 10A–C. Measurements of the scapula of PIMUZ A/V 5700, and MHNG GEPI V3659, that includes a poorly preserved distal fragment of a scapula, are given in Table 7. The scapula of *Ma. patachonica* presents a large and concave glenoid cavity (*cavitas glenoidalis*) which narrows anteriorly ending in a prominent supraglenoid tubercle (*tuberculum supraglenoidale*; also known as coracoid). Dorsal to the supraglenoid tubercle there is a wide and shallow scapular notch (*incisura scapulae*). Laterally, a strong scapular spine separates the posterior fossa infraspinata from the anterior fossa supraspinata, both being strongly concave. Although lateral-most portions of the scapular spine are missing (as in the holotype NHMUK-PVM 43402; Owen, 1838), two posterior bendings, one close to its dorsoventral midpoint and also the other one in the acromion suggest the presence of a tuber in the middle of the scapular spine (*tuber spinae scapulae*) and a caudal process of the acromion or suprahamatus process (*processus suprahamatus*). The ventral process of the acromion or hamatus process (*processus hamatus*) is small. Medially there is a marked concavity that extends dorsoventrally and it is most profound just above the supraglenoid tubercle, which we interpret as a subscapular fossa (*fossa subscapularis*) for the origin of the subscapularis muscle. Overall, the scapula of *Ma. patachonica* is very similar to other macraucheniids, such as *Theosodon* and *Xenorhinotherium* (Lessa, 1992; Scott, 1910).

The humerus of *Macrauchenia* was previously described briefly by Sefve (1925), because, in a previous contribution, he described the humerus of *Macrauchenia ullomensis* Sefvre 1914, from Ulloma, Bolivia, which he found was very similar to the humerus of *Ma. patachonica* (Sefve, 1914). However, Sefve (1914) asserted that overall, there were enough differences in the postcranial elements of both taxa to merit a species separation, which has been accepted by more recent workers (Guérin & Faure, 2004; Hoffstetter, 1986). Considering the description of Sefve (1914) and the additions of Sefve (1925), our description focuses mostly on aspects previously unnoticed. Measurements of the humerus of PIMUZ A/V 5700 are given in Table 8. The humerus of *Ma. patachonica* is overall wide but with a marked constriction close to the distal epiphysis (Fig. 10D–H). Proximally, in the posterior aspect, the humerus presents a mediolaterally wide head (*caput humeri*). In the anterior aspect of the proximal epiphysis, the medial side is dominated by a small lesser tubercle (*tuberculum minus*), whereas the lateral side is dominated by a large and anteriorly projected greater tubercle (*tuberculum majus*; Fig. 10D). The head is separated by a strong concavity from the lesser tubercle. The lesser and the greater tubercles are separated by a bicipital groove (*sulcus intertubercularis*), which in its mediolateral midpoint presents a small convexity that could be interpreted as a rudimentary intermediate tuberculum (*tuberculum intermedium*).

Anteriorly, the humerus exhibits a marked deltopectoral crest (*crista tuberculi majoris*) starting from the greater tubercle and running distally up to a moderately sized deltoid tuberosity (*tuberositas deltoidea*) laterally (Fig. 10E). Running distally from the deltoid tuberosity there is a low and subtle crest of the humerus (*crista humeri*). Medially, a low crest runs from the lesser tubercle (*crista tuberculi minoris*) up to a subtle tuberosity of the teres major (*tuberositas teres major*; Fig. 10G). Posteriorly, the tricipital line (*linea m. tricipitatis*) starts close to the midpoint between the head of the humerus and the greater tubercle and runs distolaterally ending in the deltoid tuberosity (Fig. 10F). Distal from the tricipital line, in the diaphysis, a supracondylar ridge (*crista supracondylaris lateralis*) runs laterally, starting as a low ridge and strengthening as it approaches and connects with the large lateral epicondyle (*epicondylus lateralis*) in the distal end of the humerus. Medially, the medial epicondyle (*epicondylus medialis*) is moderately developed being noticeably smaller than the lateral epicondyle. Distally, before reaching the condyle of the humerus (*condylus humeri*) there are deep fossae, the olecranon fossa (*fossa olecrani*) anteriorly, and radial fossa (*fossa radialis*) posteriorly. These two fossae do not connect through a supratrochlear foramen (*foramen supratrochleare*). Among macraucheniids, the absence of a supratrochlear foramen in *Ma. patachonica* is shared with *Theosodon* and *Xenorhinotherium* (Lessa, 1992; Scott, 1910), in contrast with *Cramauchenia* and *Llullataruca* McGrath et al., 2018, which have a marked supratrochlear foramen (Dozo & Vera, 2010; McGrath et al., 2018). In the condyle of the humerus, the capitulum (*capitulum humeri*) is markedly more distally developed than the trochlea (*trochlea humeri*), although there is no indication of separation between facets. The trochlea is somewhat anteroposteriorly thicker than the capitulum.

The specimen PIMUZ A/V 5700 preserves the ulna-radius (Fig. 10I, J) and an almost complete manus (Fig. 10K; only misses the reduced Mc V). Measurements of these elements are given in Tables 9and10. The ulna-radius and manus of *Ma. patachonica* have been described in detail in previous publications (Gervais, 1855; Owen, 1838; Parodi, 1931; Sefve, 1914, 1925). Elements of the forelimb of *Ma. patachonica* have been also recently analysed and compared with different macraucheniids that include inferences in palaeobiology and locomotion of this taxon and other members of this litoptern family (Püschel et al., 2023).

## Conclusion

We reviewed, described, and illustrated for the first time the SANU specimens from the Pampean Region of the historical Roth collection in Zurich. The collection includes two notoungulates (*Toxodon* cf. *T. platensis* and *Mesotherium cristatum*) and one litoptern (*Macrauchenia patachonica*), restricted to the “Lower” and “Intermediate” Pampean (Ensenadan to Bonaerian Stages/Ages). Although the SANUs diversity in the Roth collection is low in comparison with other groups, such as xenarthrans (Le Verger, 2023), Neartic ungulates (Carrillo-Briceño et al., 2023), and rodents (Kerber, 2023), some of the specimens are very complete, including skulls and postcranial remains. The completeness and quality of the fossil material allow us to examine and discuss previously unnoticed anatomical aspects, including new interpretations on patterns of dental wear and on the identity of the cusps present on the upper molars in *Ma. patachonica* that allow homologous comparisons with other macraucheniids and potentially other SANUs. In addition to its historical and patrimonial importance, the SANUs specimens from the Roth collection provide important information that have the potential to shed light on the study of the paleobiology and evolution of South American megafauna and evaluate hypotheses about their extinction in the continent.

## Data Availability

The data and measurements generated in this study are included in the published article.
